# Low adaptive potential for tolerance to ethynylestradiol, but also low toxicity, in a grayling population (*Thymallus thymallus*)

**DOI:** 10.1186/s12862-019-1558-1

**Published:** 2019-12-16

**Authors:** Lucas Marques da Cunha, Diane Maitre, Claus Wedekind

**Affiliations:** Department of Ecology and Evolution, Biophore, University of Lausanne, 1015 Lausanne, Switzerland

**Keywords:** Chemical pollution, Rapid evolution, Salmonidae, Estrogen, Embryo survival, Larval growth, Additive genetic variance

## Abstract

**Background:**

The presence of a novel pollutant can induce rapid evolution if there is additive genetic variance for the tolerance to the stressor. Continuous selection over some generations can then reduce the toxicity of the pollutant but also deplete the additive genetic variance for the tolerance and thereby slow down adaptation. One common pollutant that has been ecologically relevant for some time is 17alpha-ethynylestradiol (EE2), a synthetic compound of oral contraceptives since their market launch in the 1960s. EE2 is typically found in higher concentrations in rivers than in lakes. Recent experimental work revealed significant genetic variance for the tolerance to EE2 in two lake-spawning salmonid species but no such variance in river-spawning brown trout. We used another river-spawning salmonid, the European grayling *Thymallus thymallus*, to study the toxicity of an ecologically relevant concentration of EE2. We also used a full-factorial in vitro breeding design and singly rearing of 1555 embryos and larvae of 40 sib groups to test whether there is additive genetic variance for the tolerance to this pollutant.

**Results:**

We found that exposure to EE2 reduced larval growth after hatching, but contrary to what has been found in the other salmonids, there were no significant effects of EE2 on embryo growth and survival. We found additive genetic variance for embryo viability, i.e. heritability for fitness. However, there was no significant additive variance for the tolerance to EE2.

**Conclusions:**

Our findings support the hypothesis that continuous selection has reduced the toxicity of EE2 and depleted genetic variance for tolerance to this synthetic stressor.

## Background

Chemical pollution is one of the anthropogenic pressures that can threaten salmonid populations [1–3]. Salmonids are particularly exposed to such micropollutants during embryogenesis, because they typically have large eggs and long embryo developmental times, both of which enable greater uptake of ambient micropollutants during this sensitive stage [4, 5]. One of the most common pollutant is the synthetic estrogen 17α-ethynylestradiol (EE2) that is an active ingredient of most oral contraceptive pill formulations and has higher stability and estrogenic potency than its natural counterpart 17β-estradiol [6, 7]. EE2 is often detected in rivers that carry sewage treatment effluents [8, 9], and concentrations around 1 ng/L have often been measured [10]. Dissolved in water, its half-life times can be over 3 months under laboratory conditions [11], but photodegradation and the presence of co-absorbing organic matter can reduce half-life times to one or few days [12]. We therefore expect river-spawning salmonid fish to be typically exposed to higher concentrations of EE2 than lake-spawning salmonids [8, 10, 13].

Exposure to ecologically relevant concentrations of EE2 can affect gene expression in adult fish, especially in their liver and gonads [14] and in the kidneys [15]. Such EE2-induced changes affect germ cell proliferation and hormone production [14] and reduce fertility and survival of some fish [16, 17]. When applied over several years, ecologically relevant EE2 concentrations in the water can significantly change ecosystems by affecting reproduction and mean body condition in various fish [18, 19]. If these effects are sex-specific (see discussion below), population sex ratios could be affected, too [20]. Embryos and larvae may be even more susceptible to the toxicity of EE2 than adults [21]. For example, single spikes of only 2 pg EE2 added to embryos in 2 mL containers induced significant mortality and delayed hatching in two whitefish species [22]. However, such toxicity effects seem species dependent. The embryos of two other salmonid fish, the Atlantic salmon (*Salmo salar*) [23] and the brown trout (*Salmo trutta*) [11] seemed more tolerant to low concentrations of EE2, i.e. they showed lower EE2-induced mortality and lower reduction in growth (see below). These differences among salmonids are not sufficiently understood yet.

EE2 was a novel pollutant to freshwater ecosystems when the contraceptive pill was launched to the market in the 1960s. The presence of this stressor could have induced rapid evolution in some exposed salmonid populations that happened to have additive genetic variation for the tolerance to this new type of pollution [24, 25]. Continuous selection over several generations would then be expected to reduce the toxicity of EE2 but also deplete the genetic variance for its tolerance and thereby slow down adaptation [26]. If so, we would predict on average lower toxicity of, and lower genetic variation for the tolerance to, EE2 in river-spawning than in lake-spawning salmonids.

Recent experimental studies on salmonids seem to support this prediction. On the one hand, embryos of two lake-spawning salmonid species, the whitefish *Coregonus palaea* from Lake Geneva (Switzerland) and *C. albellus* from Lake Brienz (Switzerland)*,* displayed increased mortality and delayed hatching after exposure to low or high concentrations of EE2 [22]. For the lowest concentration tested in Brazzola et al. [22], a single aqueous exposure to 1 ng/L led to increases in mortalities of 3 and 13% points, respectively. Both populations also displayed significant additive genetic variance for EE2-induced embryo mortality [22]. On the other hand, embryos of two river-spawning salmonid species, the brown trout and the Atlantic salmon, showed no or weak responses to the same ecologically relevant EE2 concentration. Marques da Cunha et al. [11] found EE2 to reduce embryo survival by only 0.9% points, and they found no additive genetic variance for the tolerance to EE2 in seven genetically distinct populations. Duffy et al. [23] found no EE2-induced mortality in embryos and larvae of Atlantic salmon. They also studied vitellogenin gene transcription and plasma concentrations and found this precursor egg protein to be significantly affected only in embryos exposed to EE2 concentrations that may be too high to be ecologically relevant. However, further examples are necessary to test whether lake-spawning and river-spawning salmonids differ systematically in their reaction to EE2.

Here we focus on another river-spawning salmonid of another subfamily, the European grayling (*Thymallus thymallus*). We chose a grayling population that spawns in the River Aare in the city of Thun (Switzerland) and uses the river and the Lake Thun as feeding grounds. The population has continuously declined since the 1970s and is currently protected [27, 28]. In response to the population decline, conservation authorities have complemented their supportive breeding program based on wild-caught individuals with a broodstock based on F1 offspring from the wild population. The broodstock’s genetically effective population size (N_e_) is about a third of the wild population’s N_e_ (Marques da Cunha, Mobley, Maitre, de Guttry, Wedekind, in preparation). Because this broodstock population has been recently established and consists of F1 s only, and because population size is only weakly related to quantitative genetic variation if a population decline is recent and not too extreme [29, 30], we could avoid sampling the protected wild population and use samples from the captive population instead.

Selmoni et al. [31] found in 5 of the 40 sibgroups that are studied here (see below) that an aqueous exposure of grayling embryos to 1 ng/L EE2 caused significant changes in gene expression. These changes were strongly dependent on genetic sex and developmental stage. During the embryonic stage when whole embryos were analysed, nearly 400 genes were found to be differentially expressed in males in response to EE2, but only 15 genes in females. Around hatching and towards the end of the yolk sac period when only heads were analysed, exposure to EE2 caused differential expression of about 20,000 and 10,000 genes, respectively, with a similar number of genes being up- or downregulated. However, only females showed such strong reactions to EE2. The reactions in males were much weaker (1 and 4 genes, respectively, based on q < 0.15). New and continuous exposure to EE2 during juvenile stages then delayed the onset of sex differentiation [31], but it remained unclear whether the one-dose exposure to EE2 during the embryonic stage that induced the strong responses in the transcriptomes also reduced embryo or larval viability and growth (as in whitefish [22]) or had little effects (as in brown trout [11] and Atlantic salmon [23]).

Here we study a much larger sample and concentrate on the following questions: (i) is the toxicity of EE2 in a river-spawning grayling more comparable to the lake-spawning or to river-spawning salmonids, and (ii) is there additive genetic variance for tolerance to EE2 in the grayling population we study? High toxicity and high additive genetic variance would suggest that the population still has the potential for rapid evolution in response to this type of pollution, while high toxicity and low additive genetic variance would mean that pollution by EE2 can be one of the factors that currently contribute to the population decline [28].

## Methods

**Fig. 1:**
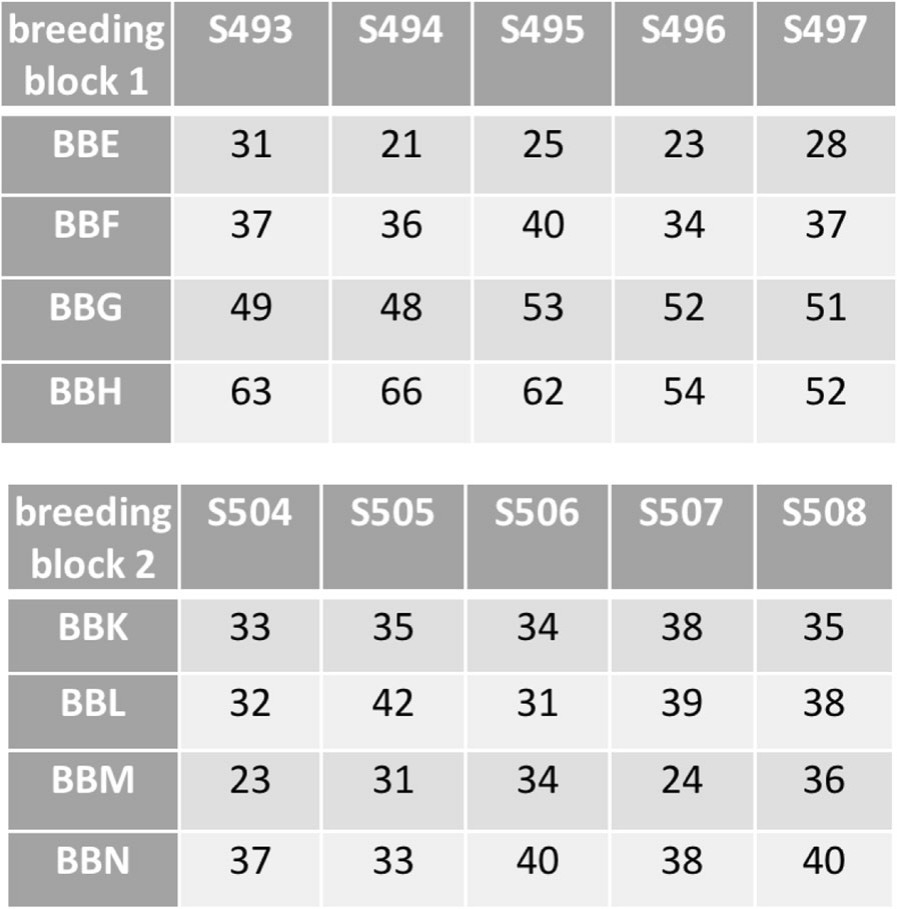
Two full-factorial breeding blocks crossing 4 females (rows) with 5 males (columns) each. Individuals were raised singly in 24-well plates until 40 dpf (day post fertilization). The figure gives the total numbers of embryos that were EE2- or control-treated

Adult grayling were sampled from a recently established captive population (cantonal *Fischereistützpunkt* Kandersteg, Bern, Switzerland) that consists of F1 of the population studied in Wedekind et al. [28]. Eight females (dams) and 10 males (sires) were stripped for their gametes and then returned to the population. These gametes were used for in vitro fertilizations in two full-factorial blocks of 4 dams × 5 sires each to produce 40 half-sib families (Fig. 1). The water used for fertilizations and the rearing of embryos was chemically standardized according to the OECD guideline No. 203 [32] and aerated before use. The freshly fertilized eggs were left undisturbed for 2 h and then transported to a climate chamber (at 7 °C) where they were washed and singly distributed to 24-well plates (Greiner Bio-One, Kremsmünster, Austria) filled with 1.8 mL of autoclaved standardized water, as in von Siebenthal et al. [33] (N_eggs_ = 10,789). After one week, eggs without visible embryo were discarded, leaving in total 7397 eggs with embryos (on average 185 embryos per sibgroup, range 89–307). These embryos were assigned to different studies: in total 250 of 5 half-sib families were used for gene expression analyses [31], another sample of 3580 embryos was exposed or sham-exposed to a pathogen to study the genetic aspects of pathogen resistance (Marques da Cunha, Mobley, Maitre, de Guttry, Wedekind, in preparation). A further sample of in total 1555 embryos was assigned to the present study (Fig. 1). After embryo and larval performance had been recorded (see below), a mixed sample of these differently treated larvae and of the remaining ones were pooled and further raised in aquaria to study sex differentiation [31, 34]. All remaining samples were euthanized with an overdose (1 mL/L) of Koimed Sleep (Ethylenglycolmonophenylether; Koimed, Ulmiz, Switzerland).

The EE2 and control stock solutions were prepared as in Marques da Cunha et al. [11]. Briefly, an EE2 stock solution of 10 ng/L made of analytical 17α-ethynylestradiol (Sigma-Aldrich, USA) and 0.004% absolute ethanol (VWR International, USA) was prepared for the EE2 treatment, and a control stock solution of 0.004% ethanol only was prepared for the sham treatment. Both stock solutions were made with autoclaved standardized water [32]. Two weeks after fertilization, 8 embryos per family were sham-treated, i.e. 0.2 mL of the control stock solution was added to each well (final water volume = 2 mL / well). All remaining embryos received 0.2 mL of the EE2 stock solution (i.e. a dose of 2 pg EE2) for a concentration of 1 ng/L and a final volume of 2 mL per well. After exposure, the embryos were regularly examined and mortality was recorded. In the last two days before the expected start of hatching (i.e., 27 and 28 days post fertilization) incubation temperature was raised from 7 °C to 10 °C and 11.5 °C, respectively, in order to reduce variance in the timing of hatching.

Each plate containing a freshly hatched larva was scanned on the day of hatching and 8 days later (Epson, Perfection V37, Japan). The larval body length and yolk sac dimensions (length and width) were measured from these scans using ImageJ (http://rsb.info.nih.gov/ij/). Yolk sac volume was calculated as described in Jensen et al. [35]. Of the in total 1347 hatchlings, 124 (9.2%) were accidently lost after hatching. These were all EE2-treated individuals from 16 of the 40 families (range 4–17 per family), i.e. each experimental cell (Fig. 1) was still well represented for the measurements after hatching. Larval growth was calculated as the difference between length after 8 days and at hatching, and yolk sac consumption as the difference between yolk sac volume at hatching and 8 days later.

Embryo and larval survival were analysed as binomial response variables in generalized linear mixed models (GLMM). Timing of hatching, hatchling length, larval growth, and yolk sac consumption were analysed in linear mixed models (LMM) as continuous response variables. Treatment and parental effects on embryo phenotypes were investigated with treatment (EE2 or control) as a fixed effect and sire and dam as random effects. Sire and dam effects are nested in breeding block, but entering breeding block as further random or fixed effect did not change any of the conclusions (results not shown). The significance of each effect was assessed by comparing models including or lacking the term of interest to a reference model. Akaike’s information criteria (AIC) were used as measures of model fit and model complexity, and likelihood ratio tests (LRT) were used to compare models. All the mixed-effects models were fitted with the lme4 R package [36] and all the statistical analyses were performed in R [37].

## Results

**Fig. 2:**
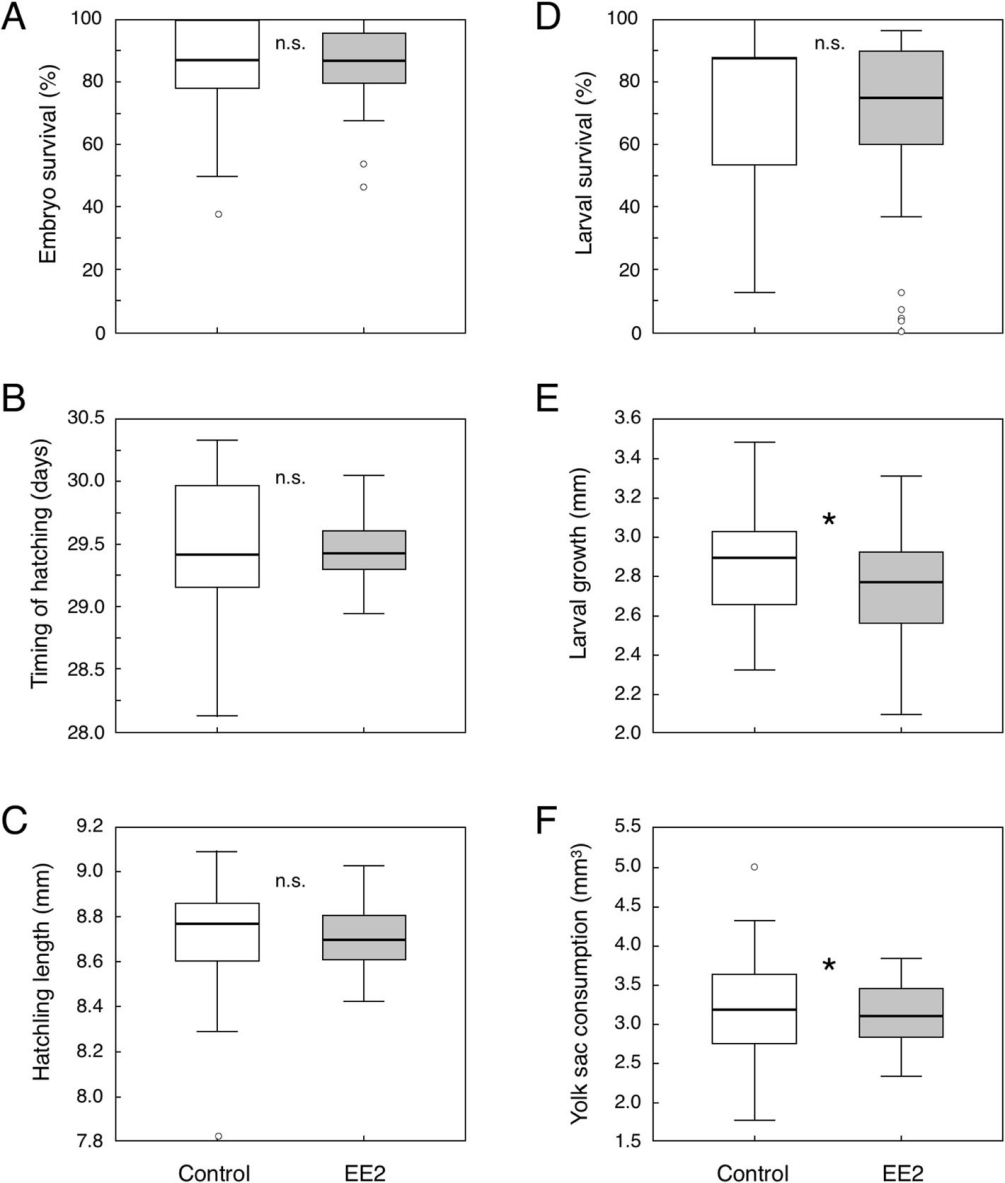
The effects of a one-dose exposure to 1 ng/L 17*α*-ethynylestradiol (EE2) on embryo and larval phenotypes: **a** embryo survival, **b** time of hatching, **c** length at hatching, **d** larval survival, **e** larval growth, and **f** yolk sac consumption during the first 8 days after hatching. Tukey outlier boxplots with quartiles, whiskers, and outliers are based on 40 family means per treatment, * = *p* < 0.05, n.s. = not significant. See Tables 1 and 2 for statistics

Total embryo survival until hatching was 86.6% (controls: 87.8%, EE2 exposed: 86.3%), and total larval survival during the first 8 days after hatching was 82.2% (controls: 82.5%, EE2 exposed: 82.1%). Maternal sib groups varied strongly in all measures of survival and growth (dam effects in Tables 1 and 2). Exposure to EE2 by itself caused no significant effects on embryo survival and growth (Table 1; Fig. 2a-c) and had no significant effects on larval survival (Table 2a; Fig. 2d). However, exposure to EE2 affected the timing of hatching differently depending on maternal sib groups (t x d interaction in Table 1b) and reduced larval growth after hatching (Table 2b, c; Fig. 2e, f).

**Table 1:** Treatment and parental effects on embryo traits. Likelihood ratio tests on mixed model regressions on (A) embryo survival, (B) timing of hatching, and (C) length at hatching. Models including or lacking the term of interest were compared to reference models (in bold) to determine the significance of the effect tested

Model	Effect tested	AIC	d.f.	χ^2^	*P*
*(A) Embryo survival*					
**t + d + s**		1166	4		
d + s	t	1165	3	0.7	0.42
t + s	d	1219	3	55.0	**< 0.001**
t + d	s	1173	3	8.8	**0.003**
t + t|d + s	t x d	1169	6	1.1	0.57
t + d + t|s	t x s	1173	6	< 0.1	1.0
*(B) Timing of hatching*					
**t + d + s**		2953	5		
d + s	t	2951	4	< 0.1	1.0
t + s	d	3038	4	86.7	**< 0.001**
t + d	s	2955	4	4.0	**0.04**
t + t|d + s	t x d	2913	7	44.7	**< 0.001**
t + d + t|s	t x s	2956	7	1.2	0.55
*(C) Length at hatching*					
**t + d + s**		1664	5		
d + s	t	1663	4	0.3	0.56
t + s	d	1688	4	25.8	**< 0.001**
t + d	s	1662	4	0.1	0.81
t + t|d + s	t x d	1664	7	3.2	0.20
t + d + t|s	t x s	1667	7	0.8	0.65

*t* treatment, *s* sire, *d* dam

Significant *p*-values are emphasized in bold

**Table 2:** Treatment and parental effects on larval traits. Likelihood ratio tests on mixed model regressions on (A) larval survival, (B) larval growth, and (C) yolk sac consumption of embryos exposed to EE2 or sham treated. Models including or lacking the term of interest were compared to reference models (in bold) to determine the significance of the effect tested

Model	Effect tested	AIC	d.f.	χ^2^	*P*
*(A) Larval survival*					
**t + d + s**		678	4		
d + s	t	679	3	2.6	0.11
t + s	d	996	3	320.2	**< 0.001**
t + d	s	678	3	2.1	0.14
t + t|d + s	t x d	680	6	2.2	0.33
t + d + t|s	t x s	682	6	0.3	0.86
*(B) Larval growth*					
**t + d + s**		778	5		
d + s	t	781	4	4.5	**0.03**
t + s	d	840	4	64.0	**< 0.001**
t + d	s	780	4	3.4	0.06
t + t|d + s	t x d	782	7	0.1	0.93
t + d + t|s	t x s	784	7	< 0.1	1.0
*(C) Yolk sac consumption*					
**t + d + s**		1479	5		
d + s	t	1481	4	4.0	**0.05**
t + s	d	1579	4	101.8	**< 0.001**
t + d	s	1477	4	< 0.1	1.0
t + t|d + s	t x d	1483	7	< 0.1	1.0
t + d + t|s	t x s	1483	7	< 0.1	1.0

*t* treatment, *s* sire, *d* dam

Significant *p*-values are emphasized in bold

Paternal sib groups differed in embryo survival, revealing additive genetic variance for fitness (Table 1a). Sire identity also affected the timing of hatching (Table 1b) but had no significant effects on larval mortality (Table 2a) nor on embryo or larval growth (Table 1c, Table 2b, c). Importantly, paternal sib groups did not significantly differ in response to EE2, i.e. there was no evidence for additive genetic variance in the tolerance to EE2 (t x s interaction terms in Tables 1 and 2).

## Discussion

The two main questions of the present study were: Is an ecologically relevant exposure to EE2 toxic to embryos and larvae of a population of river-spawning grayling, and is there additive genetic variance for the tolerance to EE2 in this population, i.e. does the population currently have a potential to rapidly adapt to this type of pollution? The first question is relevant even if the toxicity of EE2 has been demonstrated in many other fish taxa (e.g. [38–41]), because (i) the study population is declining for unknown reasons and a lack of an evolutionary response to toxicity could be contributing to the problem, and (ii) the chemical pollution of freshwater habitats that has happened since the market launch of the contraceptive pill, i.e. during more than 5 decades, could have led to adaptation and hence to reduced toxicity in some fish. The answer to the latter question may help us to better understand if pollution by EE2 has induced rapid evolution because, in our study population, the period of exposure is likely to span around 10 to 15 generations, i.e. there could have been enough time for evolution to deplete any genetic variance for tolerance to EE2 that the population could have had at the beginning of the exposure. Moreover, these questions are of ecotoxicological relevance [10, 42, 43] because standard ecotoxicological testing often ignores potential taxon-specific toxicities [44].

Regarding our first main question: We found a statistically non-significant increase in mortality of 1.5 pp. for embryos and 0.4 pp. for larvae. These effect sizes seem comparable to the observed increase in embryo mortality of 0.9 pp. in brown trout that was only significantly different from zero because of an extra-ordinary large sample size (*N* = 7302 singly raised embryos) [11]. In whitefish, the EE2-induced increase in embryos mortality was significant and around 3% points (pp) in *C. palaea* [22] and around 13 pp. in *C. albellus* [22].

With the observed low mortality, the question of whether there is EE2-induced sex-specific mortality in grayling cannot be solved yet. The study population suffers from a skewed sex ratio (more males than females [28]) that seems not due to EE2-induced sex reversal [28, 31] but rather caused by sex-specific mortality [45]. It is still possible that there are sex-specific susceptibilities to combined effects of EE2 and other environmental stressors. Other types of environmental stressors such as microbes [39], temperature variations [27], or other micropollutants [40, 46] could interact with the effects of EE2 and thereby amplify its toxicity [47, 48]. Therefore, single-factor laboratory studies like ours are likely to underestimate the ecotoxicological relevance of EE2 in the wild.

While EE2- and sham-exposed grayling embryos hatched at similar size, exposure to EE2 reduced larval growth and consumption of yolk sac after hatching by about 4% each during the first 8 days after hatching. We therefore conclude that EE2 is toxic to grayling at early developmental stages. Such a reduction in growth was predicted from recent analyses of physiological reactions to EE2 in Atlantic salmon [23, 49] but was not observed in brown trout [11]. One possible explanation for this apparent discrepancy between brown trout and grayling larvae is that hatching was not induced in the study on brown trout [11] but induced by an increase in temperature in the present study on grayling. Under the given conditions, EE2-exposed brown trout embryos hatched later and at smaller size than sham-exposed ones, while, in the present study on grayling, no treatment-related difference in the timing of hatching nor on hatchling size could be observed. If growth rate after hatching is dependent on larval size and developmental stage, such differences in the experimental protocols could be responsible for the apparent differences in treatment effects on growth rates. However, in both cases, the combined effects of EE2 on embryo and larval development would be expected to delay the emergence from gravel at the end of the yolk sac stage and could even lead to smaller body sizes at emergence. Time to emergence, and body size at emergence, is likely to be linked to fitness in salmonids because larvae that emerge earlier and larger than others may face less competition for resources (e.g. feeding territory) and are more prone to outcompete their late emerging counterparts [50, 51].

Regarding our second main question: Because grayling males do not provide any parental care, significant sire effects on offspring traits reveal additive genetic variance in full-factorial breeding experiments [26]. The dam effect then represents a combination of additive genetic variance and maternal environmental effects [26]. In salmonids, maternal environmental effects comprises characteristics such as egg size [50] and compounds that females allocate to their eggs (e.g. [52–56]). We found strong direct maternal effects on every offspring trait that we measured, and a dam x EE2 interaction on the timing of hatching. We conclude that maternal sib groups reacted differently to exposure to EE2. However, these maternal effects seem to be mainly due to maternal environmental effects [57], because we found no significant additive genetic variance for tolerance to EE2 pollution in any of the analysed traits.

No significant additive genetic variance could potentially be due to a type II error (false negative). However, such an error is unlikely here because (i) our analysis is based on a large sample size (1555 singly-reared embryos) and 40 sib groups, (ii) our sample revealed overall additive genetic variance (i.e. significant sire effects) on embryo mortality and the timing of hatching, (iii) a parallel study (Marques da Cunha, Mobley, Maitre, de Guttry, Wedekind, in preparation) on other samples of the same 40 families revealed genetic variation in the tolerance to infection by a bacterium, and (iv) singly-reared salmonid embryos are sensitive indicators of environmental stress, and studies based on comparable breeding designs have demonstrated additive genetic variance for the tolerance to other types of stressors, including other types of pollutants [58, 59], pathogens [60] or even water-borne cues linked to infection [61].

The finding of no significant additive genetic variance for tolerance to EE2 pollution in grayling is in sharp contrast to the findings of Brazzola et al. [22] on lake-spawning whitefish. However, our findings correspond well with the ones of Marques da Cunha et al. [11] who used a similar experimental protocol to test for this type of genetic variation in 7 genetically distinct populations of river-spawning brown trout and found none (in a total sample size of 7302 singly embryos, i.e. a type II error was also unlikely in their case). Taken together, these observations support the view that the appearance of the novel stressor EE2 has induced evolution and thereby used up the corresponding additive genetic variance in river-spawning salmonid that are exposed to the pollutant, while lake-spawning salmonids who are less exposed still have a strong potential to evolve rapidly to EE2. However, alternative explanations are possible. Future studies could therefore compare exposed and non-exposed populations of the same species (if at all possible, given human population density and the finding that very low doses of EE2 can induce selection), add analogous tests on further river-or lake-spawning salmonids, or test for signatures of selection in the EE2 response pathways [62, 63].

As far as we know, there exist no measurements of estrogenic pollution around the spawning ground of our study population. However, this spawning ground is located in the river Aare within a city of more than 40,000 inhabitants, a large sewage treatment plant about 4 km downstream, and several nearby villages (with several thousand inhabitants each) upstream. The sewage treatment process typically removes only about two thirds of the EE2 [8], and exposure to EE2 is therefore likely in rivers of the Swiss Plateau [8, 64]. Marques da Cunha et al. [11] sampled brown trout from 7 different streams (the river Aare and 6 tributaries) to test whether variation in estrogenic pollution creates population differences in toxicity of EE2. They found population differences in various embryo and larval traits, but none in the reaction to EE2. They argued that very low concentrations on EE2 and exposure during only short periods can cause selection and hence induce rapid evolution. The hypothesis is supported by the observation that the 2 pg EE2 in the aqueous exposure seemed to be continuously taken up by the embryo (about 80% within 4 weeks) while the concentration remained constant in empty plates [11]. This suggests that salmonid eggs take up EE2 at concentrations that are far lower than the 1 ng/L that are sometimes even found in groundwater [65]. On the other side of the scale: when Brazzola et al. [22] exposed whitefish embryos to 1 ng/L, 10 ng/L, or 100 ng/L EE2, increasing concentration seemed only weakly linked to increased toxicity. Similar observations were made by Duffy et al. [23] who exposed Atlantic salmon to 1.2 ng/L, 11.9 ng/L, and 118.6 ng/L EE2, respectively. We therefore argue that our one-dose aqueous exposure to 2 pg EE2 was ecologically relevant for grayling embryos and likely to reveal additive genetic variance for tolerance, should it exist.

Our study adds the grayling to the list of salmonids whose embryos and larvae could be experimentally exposed to ecologically relevant concentrations of around 1 ng/L EE2. With the present study, at least one species of each subfamily of the Salmonidae (Coregoninae, Salmoninae, and Thymallinae) has now even been tested using the same method of applying a one-dose exposure of 2 pg to embryos developing in 2 mL wells [11, 22]. Together, these studies reveal strong species-specific reactions to EE2 within the salmonids, and various amounts of additive genetic variance in the tolerance to this synthetic stressor.

## Conclusions

A key question in evolutionary conservation biology is whether populations can adapt to anthropogenic stressors such as chemical pollutants. Such evolutionary responses require additive genetic variance for the susceptibility to the pollutant. Continuous selection over several generations is then expected to deplete such genetic variance but also reduce the toxicity of the pollutant. We tested the susceptibility of a river-spawning grayling population to EE2 and found that a low and ecologically relevant concentration did not induce embryo mortality as it did in some lake-spawning salmonids. However, EE2 was still toxic because it reduced larval growth. We found additive genetic variance for fitness-relevant traits, but no significant genetic variation that would enable the population to adapt to EE2. The low toxicity and the lack of genetic variance for the susceptibility to EE2 support the hypothesis that the marked launch of contraceptive pills and the associated pollution of rivers has induced rapid evolution in river-spawning grayling.

## Data Availability

The data used in this study are available from the Dryad Digital Repository: 10.5061/dryad.7wm37pvp0

## Abbreviations

AIC: Akaike’s information criteria
EE2: 17alpha-ethynylestradiol
GLMM: Generalized linear mixed model
LMM: Linear mixed model
LRT: Likelihood ratio test
N_e_: Genetically effective population size
